# ABCC1 Is a ΔNp63 Target Gene Overexpressed in Squamous Cell Carcinoma

**DOI:** 10.3390/ijms25168741

**Published:** 2024-08-10

**Authors:** Veronica La Banca, Sara De Domenico, Sara Nicolai, Veronica Gatti, Stefano Scalera, Marcello Maugeri, Alessandro Mauriello, Manuela Montanaro, Jens Pahnke, Eleonora Candi, Silvia D’Amico, Angelo Peschiaroli

**Affiliations:** 1 Department of Experimental Medicine, University of Rome “Tor Vergata”, Via Montpellier 1, 00133 Rome, Italy; labanca.veronica@gmail.com (V.L.B.); sara.dedomenico95@gmail.com (S.D.D.); alessandro.mauriello@uniroma2.it (A.M.); candi@uniroma2.it (E.C.); 2 Institute of Translational Pharmacology (IFT), CNR, Via Fosso del Cavaliere 100, 00133 Rome, Italy; sara.nicolai@cnr.it (S.N.); v.gatti@unicampus.it (V.G.); 3 UOSD Clinical Trial Center, Biostatistics and Bioinformatics Division, IRCCS Regina Elena National Cancer Institute, 00144 Rome, Italy; stefano.scalera@ifo.it (S.S.); marcello.maugerisacca@ifo.it (M.M.); 4 Department of Biomedicine and Prevention, University of Rome “Tor Vergata”, Via Montpellier 1, 00133 Rome, Italy; manuela.montanaro@uniroma2.it; 5 Translational Neurodegeneration Research and Neuropathology Lab/Section of Neuropathology Research, Department of Pathology (PAT), Medical Faculty/Clinical Medicine (KlinMed), Clinics for Laboratory Medicine (KLM), University of Oslo (UiO) and Oslo University Hospital (OUS), Sognsvannsveien 20, 0372 Oslo, Norway; jens.pahnke@gmail.com; 6 Institute of Nutritional Medicine (INUM)/Lübeck Institute of Dermatology (LIED), University of Lübeck (UzL) and University Medical Center Schleswig-Holstein (UKSH), Ratzeburger Allee 160, D-23538 Lübeck, Germany; 7 Department of Pharmacology, The Faculty of Medicine and Life Sciences, University of Latvia (LU), Jelgavas iela 3, LV-1004 Rīga, Latvia; 8 Department of Neurobiology, School of Neurobiology, Biochemistry and Biophysics, The Georg S. Wise Faculty of Life Sciences, Tel Aviv University (TAU), Tel Aviv 6997801, Israel; 9 Biochemistry Laboratory, Istituto Dermopatico Immacolata (IDI-IRCCS), 00166 Rome, Italy

**Keywords:** epidermal differentiation, skin inflammation, p63, ABC transporter

## Abstract

The transcription factor ΔNp63 plays a pivotal role in maintaining the integrity of stratified epithelial tissues by regulating the expression of distinct target genes involved in lineage specification, cell stemness, cell proliferation and differentiation. Here, we identified the ABC transporter subfamily member *ABCC1* as a novel ΔNp63 target gene. We found that in immortalized human keratinocytes and in squamous cell carcinoma (SCC) cells, ∆Np63 induces the expression of ABCC1 by physically occupying a p63-binding site (p63 BS) located in the first intron of the *ABCC1* gene locus. In cutaneous SCC and during the activation of the keratinocyte differentiation program, ∆Np63 and ABCC1 levels are positively correlated raising the possibility that ABCC1 might be involved in the regulation of the proliferative/differentiative capabilities of squamous tissue. However, we did not find any gross alteration in the structure and morphology of the epidermis in humanized *hABCC1* knock-out mice. Conversely, we found that the genetic ablation of *ABCC1* led to a marked reduction in inflammation-mediated proliferation of keratinocytes, suggesting that ABCC1 might be involved in the regulation of keratinocyte proliferation upon inflammatory/proliferative signals. In line with these observations, we found a significant increase in ABCC1 expression in squamous cell carcinomas (SCCs), a tumor type characterized by keratinocyte hyper-proliferation and a pro-inflammatory tumor microenvironment. Collectively, these data uncover *ABCC1* as an additional ∆Np63 target gene potentially involved in those skin diseases characterized by dysregulation of proliferation/differentiation balance.

## 1. Introduction

The transcription factor p63, the most ancient member of the p53 family, is a master regulator of epithelial development and homeostasis [1,2]. Numerous protein isoforms with distinct characteristics are produced by its encoding gene *TP63*, such as the full-length TAp63 and the N-terminally truncated ΔNp63 [3]. The most prevalent isoform expressed in the basal layer of stratified epithelia is ΔNp63, whose transcriptional activity is pivotal to preserve the proliferative potential of epithelial basal cells in stratified epithelia, including skin, skin appendages, mammary gland and thymus [4,5,6]. Numerous studies have shown how important p63 is for epithelial formation. The development of many epithelial tissues, including thymus, breast, and epidermis, is severely hindered by the genetic deletion of all p63 isoforms, leading to the premature death of the newborn mice due to severe dehydration [7,8]. Interestingly, the p63 global null-mouse phenotype is recapitulated by the selective genetic deletion of the ΔNp63 isoforms, strongly suggesting that ΔN variants are required for appropriate epithelial structure formation [9]. At the molecular level, ΔNp63 can act as transcriptional activator or repressor towards diverse target genes involved in regulating stem cell function, cell adhesion and, paradoxically, activating the differentiation program [4,10,11,12,13]. For instance, ΔNp63 can inhibit cell cycle arrest genes, such as p21, sustaining cell cycle progression in the basal layer of the skin [14], and at the same time induces the expression of ZNF750 and JAG2, favoring the epidermal differentiation [15,16]. ΔNp63-dependent transcriptional activity relies on its ability to recruit distinct epigenetic modulators and chromatin remodeling complexes, thus affecting the epigenetic landscape of epithelial cells [17,18,19,20]. For example, our recent study unveiled that in proliferating keratinocytes, ΔNp63 recruits the histone deacetylase HDAC1 to the proximal promoter of the *NEAT1* genomic locus, thereby repressing the expression of the lncRNA NEAT1 [21].

Being a master gene controlling epithelial cell fate, it is not surprising that ΔNp63 transcriptional activity and expression are dysregulated in human diseases affecting epithelial integrity, including epithelial tumors [22,23,24]. In detail, *TP63* is frequently amplified or overexpressed in squamous cell carcinomas (SCCs) of the head and neck, skin, lung and esophagus [25]. Furthermore, additional genetic events, such as *NOTCH* mutations, IRF6 down-modulation, *ACTL6a* and *SOX2* amplification may foster ΔNp63 oncogenic activity in SCCs [26,27,28,29]. In SCCs, ΔNp63 controls specific oncogenic programs related to apoptosis resistance and promotion of growth, drug resistance, cell migration and invasion, and growth factor signaling [23,30,31,32,33]. We have previously demonstrated that the modulation of the hyaluronic acid (HA) metabolism and signaling by ∆Np63 impacts the expression of ABCC1/MRP1 [31,32], an ABC transporter subfamily member capable of regulating the extracellular efflux of various endogenous metabolites and xenobiotics across cellular membranes [34,35,36]. Here, we further investigated the regulation of *ABCC1* gene expression by ΔNp63 by providing the molecular details of ΔNp63-dependent regulation of ABCC1 as well as its impact on epidermis homeostasis.

## 2. Results

### 2.1. ΔNp63 Regulates the Expression of ABCC1

**Figure 1:**
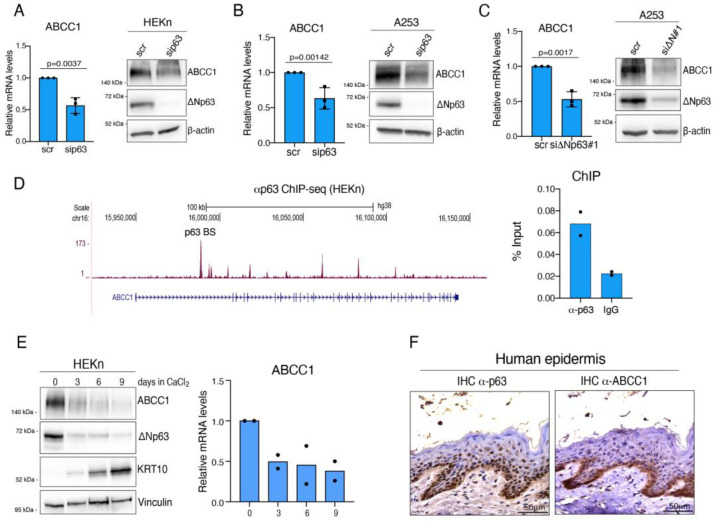
(**A**) RT-qPCR analysis (**left** panel) of ABCC1 mRNA levels in human primary keratinocytes (HEKn) transfected with siRNA oligos targeting p63 (sip63) or non-relevant mRNA. The mean of three (*n* = 3) independent biological replicates ± SD is shown. *p* value was calculated using two-tailed unpaired Student’s *t* test. In parallel, Western blotting analysis using antibodies to the indicated proteins (right panel) was performed utilizing protein lysates from transfected cells. (**B**) Human HNSCC A253 cell line was transfected and analyzed as described in (**A**). (**C**) RT-qPCR analysis (**left** panel) of ABCC1 mRNA levels in A253 transfected with siRNA targeting ΔNp63 isoform (siΔNp63#1) or non-relevant mRNA (scr). The mean of three (*n* = 3) independent biological replicates ± SD is shown. *p* value was calculated using two-tailed unpaired Student’s *t* test. In parallel, Western blotting analysis using antibodies to the indicated proteins (**right** panel) was performed utilizing protein lysates from transfected cells. (**D**) ChIP-qPCR analysis (**right** panel) of endogenous ΔNp63 binding to the *ABCC1* genomic locus. p63 binding region was determined by analyzing publicly available p63 ChIP-seq data obtained in HEKn cells (GSM1446927) (**left** panel: p63 binding sites in purple; *ABCC1* gene locus in blue). Average values from *n* = 2 biological replicates measured using three technical replicates are plotted. (**E**). Western blotting (**left** panel) or RT-qPCR analysis (**right** panel) was performed utilizing protein lysates or total RNA extracted from HEKn at different time points (0, 3, 6, 9 days) upon CaCl_2_ treatment. The mean of two (*n* = 2) independent biological RT-qPCR replicates is shown. (**F**) Representative images of immunohistochemical analysis of p63 and ABCC1 expression in normal skin samples.

Our group has previously reported that in HNSCC, the ∆Np63-dependent regulation of hyaluronic acid (HA) impacts chemosensitivity, likely through the ABC transporter ABCC1 [31], suggesting the existence of a functional link between ∆Np63 transcriptional activity and ABCC1 expression. To test this hypothesis, we used a siRNA-mediated approach to deplete p63 expression in human primary keratinocytes (HEKn) and in the A253 squamous cell carcinoma (SCC) cell line. We found that p63 depletion decreases the expression of ABCC1 at both the mRNA and protein levels (Figure 1A,B). HEKn and A253 cells exclusively express the ∆Np63 isoform [31], suggesting that this isoform might be responsible for the modulation of ABCC1 expression. Accordingly, we found that the specific depletion of the ∆Np63 isoform with two different siRNA oligos markedly decreases ABCC1 mRNA and protein levels in A253 cells (Figure 1C and Figure S1).

To study the molecular mechanism underlying the ∆Np63-dependent regulation of ABCC1 expression, we tested whether ∆Np63 can bind to the *ABCC1* genomic locus. To this end, we performed a chromatin immunoprecipitation (ChIP) experiment in HEKn cells, utilizing publicly available ChIP-seq data for these primary cells (Figure 1D, left panel). We found that endogenous ∆Np63 occupies a p63 binding site (p63 BS) located in the first intron of the *ABCC1* gene locus (Figure 1D, right panel). Altogether, these data indicate that ABCC1 is a direct transcriptional target gene of ∆Np63.

### 2.2. ABCC1 Expression Is Modulated during Keratinocyte Differentiation

During epidermal differentiation, the expression levels of ∆Np63 are tightly regulated, being high in proliferating keratinocytes located in the basal layer of the skin and decreasing in the super-basal layers, coinciding with the activation of the epidermal differentiation program [37]. To test whether ABCC1 expression undergoes a differentiation-dependent modulation similar to that of ∆Np63, we analyzed its expression in the well-established model of calcium-induced differentiation of primary human keratinocytes in vitro. We found that ABCC1 and ∆Np63 levels are similarly downregulated during epidermal differentiation, concomitant with upregulation of the differentiation marker keratin 10 (KRT10) (Figure 1E). To confirm this result in vivo, we performed an immunohistochemical analysis on human skin samples. As shown in Figure 1F, ∆Np63 and ABCC1 are mainly localized in the basal compartment, and their protein levels decrease in the upper layer of the human epidermis, indicating that ∆Np63-dependent regulation of ABCC1 expression occurs in a physiological context in which ∆Np63 is critically involved.

### 2.3. ABCC1 Deletion Does Not Impair Skin Development

**Figure 2:**
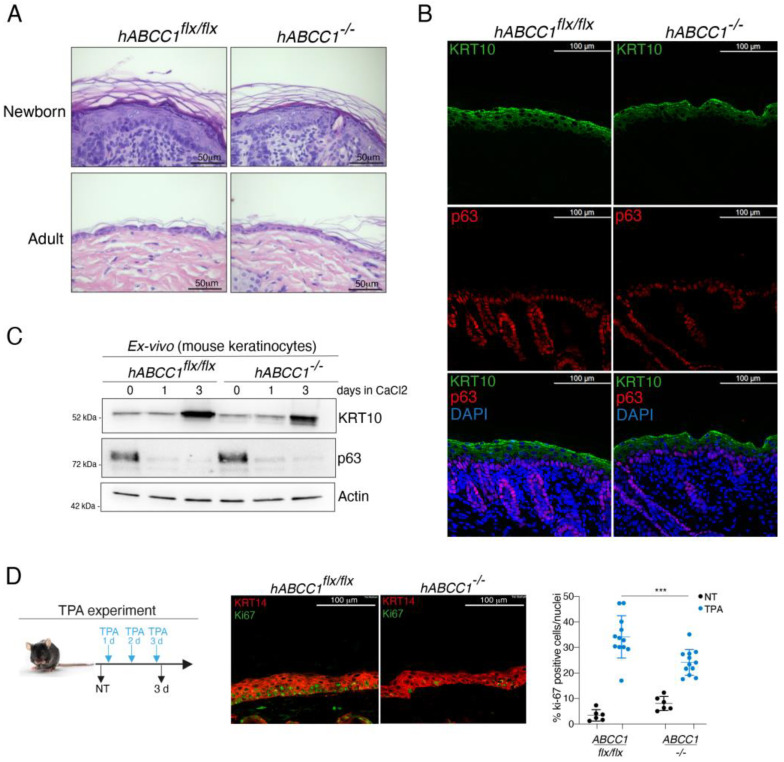
(**A**) Representative images of Hematoxylin/eosin (H&E) staining of newborn and adult epidermis of *hABCC1^flx/flx^* and *hABCC1^−/−^* mice. (**B**) Immunofluorescence staining of the differentiation marker KRT10 (green) and the basal marker p63 (red) in epidermis of *hABCC1^flx/flx^* and *hABCC1^−/−^* mice. DAPI staining (blue) was used to visualize the nuclei. (**C**) Immunoblot analysis of the indicated proteins in murine keratinocytes isolated from the epidermis of *hABCC1^flx/flx^* and *hABCC1^−/−^* mice. (**D**) TPA treatment as indicated in the left panel was performed in the dorsal skin of *hABCC1^flx/flx^* and *hABCC1^−/−^* mice. Representative images of the immunofluorescence analysis of epidermis isolated from the TPA-treated mice (central panel). Keratin 14 (KRT14) (red) is a marker of the basal layer, while Ki67 staining (green) is utilized to visualize proliferating cells. Quantification of Ki67 positive basal cells in *hABCC1^flx/flx^* and *hABCC1^−/−^* TPA-treated epidermis (right panel). Each dot represents the count of Ki67 positive basal cells over the DAPI positive cells in each microscopic field. We analyzed *n* = 6 microscopic fields (*n* = 6) for each mouse (*n* = 2). Data shown are the mean of *n* = 12 measurements ± SD for *hABCC1^flx/flx^* and *hABCC1^−/−^* TPA-treated epidermis. *** *p* value < 0.001. *p* value was calculated using two-tailed unpaired Student’s *t* test.

The results described above indicate that ABCC1 expression, like ∆Np63, is modulated during epidermal differentiation, raising the possibility that ABCC1 might be involved in the regulation of the proliferative/differentiative capabilities of human keratinocytes. To test whether ABCC1 depletion affects keratinocyte proliferation, we performed the proliferation assay in human primary keratinocytes (HEKn) upon ABCC1 depletion utilizing two different ABCC1 targeting siRNA oligos (siABCC1#1 and siABCC1#2). As shown in the Figure S2, we observed a slight decrease in keratinocyte proliferation upon ABCC1 silencing, suggesting that ABCC1 depletion may to some extent impact keratinocytes proliferation in cultured cells. To validate this data in vivo, we studied the effect of the genetic deletion of *ABCC1* on skin morphogenesis, which is the result of a highly regulated balance between proliferation and differentiation of basal keratinocytes. We analyzed the skin morphology of wild-type (*hABCC1^flx/flx^*) and *hABCC1* knock-out (*hABCC1^−/−^*) mice. The *hABCC1^flx/flx^* is an ABCC1 humanized knock-in mouse model in which the murine *Abcc1* gene has been replaced with the human ortholog (*hABCC1*) [31]. We performed H&E staining of the skin derived from newborn and adult wild-type and *hABCC1^−/−^* mice. We did not observe any gross alterations in the structure and morphology of the epidermis upon hABCC1 genetic deletion (Figure 2A). Accordingly, immunofluorescence staining of *hABCC1^−/−^* epidermis did not reveal any changes in the expression and localization of the differentiation marker KRT10 and the basal marker p63 (Figure 2B). To further corroborate these results, we performed an ex vivo analysis of murine keratinocytes derived from wild-type and *hABCC1^−/−^* mice. Consistently with the immunofluorescence study, we did not observe any significant changes in the expression of KRT10 and ∆Np63 in *hABCC1^−/−^* keratinocytes (Figure 2C). These data indicate that in vivo *ABCC1* deletion seems to not affect the proper activation of the epidermal differentiation program or the proliferative capabilities of keratinocytes.

Since ABCC1 controls the extracellular efflux of pro-inflammatory lipids, such as prostaglandin LCT4, which are involved in the regulation of cell proliferation in response to inflammatory signals [32], we tested whether *ABCC1* deletion impacts the cellular response to the pro-inflammatory agent 12-O-tetradecanoylphorbol-13-acetate (TPA), which induces skin inflammation and keratinocyte proliferation. As shown in Figure 2D, the genetic ablation of *ABCC1* led to a marked reduction in TPA-mediated proliferation, suggesting that ABCC1 might be involved in the regulation of keratinocyte proliferation upon inflammatory/proliferative signals.

### 2.4. ABCC1 and p63 Protein Levels Are Increased in SCC

**Figure 3:**
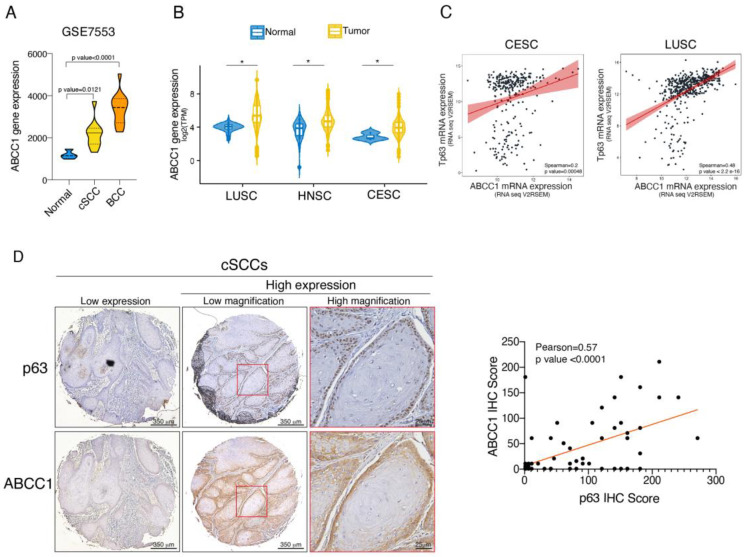
(**A**) Violin plot illustrating ABCC1 expression values in basal cell carcinomas (BCC, *n* = 15), cutaneous squamous cell carcinomas (cSCC, *n* = 11) and in the respective normal tissues (normal). *p* value was calculated using two-tailed unpaired Student’s *t* test. (**B**) Violin plot illustrating ABCC1 expression values in lung SCC (LUSC, *n* = 466), head and neck SCC (HNSC, *n* = 517), cervical SCC (CESC, *n* = 303) and the respective normal tissues. * indicates *p* value < 0.05. (**C**) Co-expression analysis of TP63/ABCC1 in datasets of human cervical SCC (CESC) (TCGA, provisional) and human lung SCC (LUSC) (TCGA, provisional) was performed at cBioPortal for Cancer Genomics (http://www.cbioportal.org accessed on 28 May 2024). Spearman correlation coefficient is reported. (**D**) Representative images of immunohistochemistry analysis of p63 and ABCC1 expression in cSCC (left panel). Red box indicates area of higher magnification image. Correlation plot of ABCC1 and p63 IHC-scores in 70 human cSCC tumor samples (right panel). *p* value (*p*) of the Pearson’s correlation coefficient (0.57) is reported.

Squamous cell carcinoma (SCC) is a highly malignant tumor characterized by dysregulation of a subset of functionally related genes regulating the balance between proliferation and differentiation [25,38,39]. Based on the observation that ABCC1 expression might be linked to the aberrant proliferation of keratinocytes, we analyzed ABCC1 expression in basal cell carcinoma (BCC) and cutaneous SCC (cSCC), two skin diseases characterized by keratinocyte hyper-proliferation [40,41]. By analyzing probes matching ABCC1 mRNA in a GEO microarray dataset including 15 basal cell carcinoma (BCC) and 11 cSCC tissue samples, we observed a significant increase in ABCC1 expression in both BCC and cSCCs (Figure 3A). By analyzing TCGA datasets, we validated the increased expression of ABCC1 mRNA levels in additional SCCs, including lung (LUSC), head and neck (HNSC), and cervical (CESC) (Figure 3B). Since different SCCs are commonly characterized by increased expression and enhanced activity of ∆Np63, we tested whether p63 and ABCC1 levels are positively correlated in SCCs. As shown in Figure 3C, we found that p63/ABCC1 co-expression is highly correlated in LUSC and, to a minor degree, in CESC. To validate this observation at protein levels, we performed immunohistochemistry (IHC) staining of ABCC1 and p63 in a cSCC tissue microarray (TMA). As shown in Figure 3D, we observed a correlation between p63 and ABCC1 protein levels in 70 tumor samples. Collectively, these data suggest that although ABCC1 is not required for maintaining the integrity and homeostasis of epidermal tissues, its dysregulation is associated with skin diseases characterized by unbalanced proliferation/differentiation pathways.

## 3. Discussion

**Figure 4:**
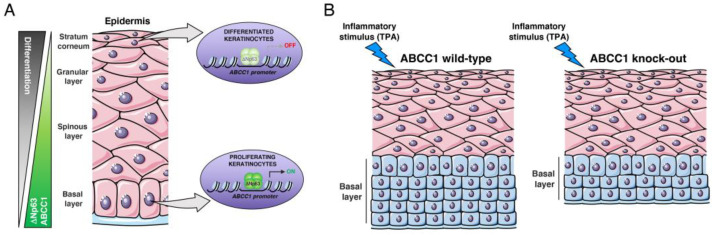
(**A**) Schematic representation of the ΔNp63-dependent regulation of ABCC1 expression during epidermal differentiation. (**B**) The genetic ablation of ABCC1 led to a marked reduction in inflammation-driven proliferation of basal keratinocytes (see text for details). The figure was drawn in part using and/or modifying images from Servier Medical Art. Servier Medical Art is licensed under CC BY 4.0 (https://creativecommons.org/licenses/by/4.0/ accessed on 28 May 2024).

ABCC1, also known as multidrug resistance-associated protein 1 (MRP1), is a member of the ATP-binding cassette (ABC) transporter subfamily, which is involved in transporting various endogenous metabolites and xenobiotics across cellular membranes [34,35,36,42]. Given its role in promoting the extracellular efflux of diverse chemotherapeutic drugs, such as daunorubicine, etoposide and camptothecine, it is not surprising that the most studied function of ABCC1 in human pathology is related to its capacity to induce chemoresistance in tumor cells [43,44,45,46]. Forced expression of ABCC1 confers chemoresistance in various tumor cell types, such as breast carcinoma and colon carcinoma [43,47]. Consistent with this evidence, ABCC1 expression is elevated in many hematopoietic and solid tumors [43]. In HNSCC, three studies reported high levels of ABCC1 in a subset of HNSCC patients [48,49,50]. In one of these studies, high expression of ABCC1 was associated with poor prognosis in patients with oral carcinoma treated with radiotherapy and chemotherapy, suggesting that ABCC1 expression might be clinically relevant [50]. Generally, the increased expression of ABCC1 in human tumors is the result of transcriptional activation or gene amplification [51]. Accordingly, well-established oncoproteins have been reported to act as transcriptional regulators of *ABCC1*. For instance, c-jun/junD complexes, MYC, and NRF2 bind to specific responsive elements in the *ABCC1* promoter [52,53,54]. In this manuscript, we identified the transcription factor ΔNp63 as an additional player involved in ABCC1 gene expression regulation (see Figure 4).

ΔNp63 plays a crucial role in the biology of stratified epithelia, and its transcriptional activity regulates the proliferative potential and stemness of basal epithelial cells. We found that in primary human keratinocytes and SCC cells, ∆Np63 positively regulates the expression of ABCC1 by physically occupying a p63 binding site (p63 BS) located in the first intron of the *ABCC1* gene locus. The ∆Np63-dependent regulation of ABCC1 expression is consistent with the observation that SOX2, another transcription factor involved in squamous epithelia homeostasis, transactivates *ABCC1* promoter [55]. Furthermore, SOX2 and p63 are co-expressed in the stem/progenitor cell compartments of epithelial tissues as well as in SCC cells, where they exhibit overlapping genomic occupancy at numerous loci [56]. These observations suggest that ∆Np63 and SOX2 might act in concert to sustain the expression of ABCC1 to regulate the stemness capability of normal and tumor tissues. In agreement with this concept, we found that ABCC1, similarly to ∆Np63, is exclusively localized to the basal compartment of the human epidermis, where stem/progenitor cells reside. Notably, ABCC1 protein levels decrease in the upper layers of the human epidermis, coinciding with the activation of the epidermal differentiation program and the loss of stemness properties. Given that ∆Np63 levels are high in proliferating keratinocytes and decrease in the upper layers of the human epidermis, where differentiation occurs [37], it is possible that ∆Np63 downmodulation might favor the concomitant downregulation of ABCC1 together with the activation of epidermal differentiation. However, we cannot rule out that other mechanisms, such as control of ABCC1 stability, could contribute to the ABCC1 modulation during skin differentiation. The exclusive localization of ABCC1 in the basal layer of the epidermis may exert various biological functions. As a regulator of GSH/GSSG efflux [57], ABCC1 might help in maintaining the redox homeostasis, thereby contributing to the stem-like properties of basal cells. By facilitating the transport of xenobiotics [42], ABCC1 could protect the basal epithelial cells from the harmful effects of exogenous insults. Since its expression is downmodulated during keratinocyte differentiation, ABCC1 could regulate the balance [37] between proliferation and differentiation, acting as a modulator of keratinocyte differentiation.

Although our siRNA studies suggest some effect on keratinocyte proliferation, our in vivo data indicate that ABCC1 loss does not impact the formation of epidermis. Furthermore, the in vivo loss of ABCC1 does not affect the expression and localization of ΔNp63, which acts as a master regulatory gene of keratinocyte proliferation. It is possible that under physiological conditions, compensatory mechanisms exerted by other ABC family members maintain normal skin development in *hABCC1 KO* mice or that ABCC1 activity is important to regulate skin homeostasis in response to specific stimuli or insults. Accordingly, we found that the genetic ablation of ABCC1 led to a marked reduction in the basal keratinocyte proliferation induced by inflammatory/proliferative signals. As an important transporter of pro-inflammatory biolipids such as leukotriene LTC4 and prostaglandins [42], ABCC1 transporter activity might sustain the inflammatory microenvironment, thereby promoting cell proliferation in response to inflammatory signals. In line with this hypothesis, we observed a significant increase in ABCC1 expression in different SCCs, including skin, head and neck and cervical SCCs, which are characterized by keratinocyte hyper-proliferation and a pro-inflammatory tumor microenvironment [58,59]. Furthermore, in a panel of 70 cSCC tumor samples and in CESC and LUSC datasets, we observed a strong correlation between p63 and ABCC1 expression, strengthening the functional link between these two proteins. Although we did not explore the specific role of ABCC1 in squamous carcinogenesis, we can speculate that it is not merely related to chemoresistance.

Indeed, robust and convincing evidence of a role for ABCC1 in modulating chemoresistance during in vivo squamous carcinogenesis is lacking. Most evidence is based on overexpression studies performed in tumor cell lines, and evidence that ABCC1 chemical inhibition or its genetic deletion increases chemosensitivity in animal models of squamous carcinogenesis is missing. Based on its role in modulating the export of diverse endogenous metabolites, ABCC1 targeting might impact other tumor-related phenomena that are not directly linked to its ability to modulate the extracellular efflux of anti-neoplastic agents. For instance, the pro-tumorigenic action of ABCC1 might be also linked to its ability to promote the efflux of pro-inflammatory signals, which may shape the immune landscape to support the growth and response to immunotherapy of tumor cells. This hypothesis could be relevant in SCCs, which are characterized by a highly inflamed tumor microenvironment [58,59]. These considerations, together with the development of selective and high-affinity ABCC1 inhibitors, could provide novel ABCC1-based therapeutic approaches for targeting tumors with elevated expression of ABCC1, such as SCC.

Collectively, our data characterize ABCC1 as a novel ∆Np63 target gene potentially involved in those skin diseases characterized by alterations in the proliferation/differentiation balance and/or driven by inflammatory signals. Further research is needed to fully elucidate the relevance of ∆Np63–ABCC1 functional interaction in skin physiology and pathology.

## 4. Materials and Methods

### 4.1. Cell Culture and Transfection

A253 cells (ATCC HTB-41) were grown in McCoy’s medium (Gibco, Invitrogen, Waltham, MA, USA) with the addition of 10% fetal bovine serum (FBS), 100 μg/mL penicillin and 100 μg/mL streptomycin (Gibco, Invitrogen). HEKn cells (neonatal normal human epidermal keratinocytes, Life Technologies, Carlsbad, CA, USA) were cultured in EpiLife medium plus growth supplements (HKGS, Life Technologies, Carlsbad, CA, USA). HEKn differentiation was induced by adding 1.2 mM CaCl_2_ to the culture medium of sub-confluent cells. All the cells were maintained at 37 °C with 5% CO_2_. For p63 siRNA-mediated knockdown, HEKn and A253 cells were transfected with the following specific siRNAs: sip63 5′-CAGGUUGGCACUGAAUUCA-3′, siΔNp63#1 5′-GAAGAAAGGACAGCAGCATTG-3′, siΔNp63#2 5′-ACAAUGCCCAGACUCAAUUUU3′ and nonrelevant siRNA (scr), purchased from Sigma-Aldrich. For ABCC1-mediated knockdown, HEKn cells were transfected with the following specific siRNAs: siABCC1#1 5′-CAUUGCAGGUCACCACGUA-3′ and siABCC1#2 5′ CUCUCUACCUCCUGUGGCU-3′, purchased from Sigma-Aldrich. All transfections were performed using the Lipofectamine RNAiMAX transfection reagent (Invitrogen, Waltham, MA, USA) according to manufacturer’s protocols.

### 4.2. Growth Curve Analysis

HEKn cells were seeded into 10 mm plates (1 × 10^6^ cells) and allowed to adhere overnight. The next day, cells were transfected with nonrelevant siRNA (SCR) or siRNA targeting two different regions of ABCC1 mRNA (siABCC1#1 or siABCC1#2). At 24 h post transfection, cells were trypsinized and re-seeded into 6-well plates, ensuring four replicates for each experimental condition. The growth curve of the transfected cells was assessed over a period of 72 h. Cell counts were performed at 24-hour intervals (0 h, 24 h, 48 h, and 72 h). At each time point, cell viability and number were determined using the trypan blue exclusion method.

### 4.3. Animal Studies

Mice were maintained in the animal facility of the University of Rome “Tor Vergata” under approved conditions. The ABCC1 humanized knock-in (*hABCC1^flx/flx^*) and knockout (*hABCC1^−/−^*) mouse models (C57Bl/6J background) were obtained from our collaborator Prof. Jens Pahnke (University of Oslo) [60]. The crossbreeding of *hABCC1^flx/flx^* mice with Cre-deleter mice resulted in the global deletion of *hABCC1*. Males/females were equally distributed. All mouse procedures were carried out in accordance with institutional standard guidelines and under the authorization of the Italian Health Minister.

### 4.4. Keratinocyte Isolation Assay

Newborn mouse skins were isolated and incubated with the dermis side down in 0.25% Trypsin-EDTA (Gibco, Waltham, MA, USA, cat. num. 25200056) overnight at 4 °C. After enzymatic digestion, the epidermises were separated from the dermises and minced for approximately 5 min using sterile scissors or scalpels. The dissociated tissues were collected with Low calcium medium (EMEM Lonza, Portsmouth, NH, USA, cat. num. 06-174G), pooled and filtered using a 100 μm Cell Strainer (BD Falcon, Dhaka, Bangladesh, cat. num. 352360) to remove corneocytes, and centrifuged at 800 rpm for 8 min. The cell pellet was then resuspended in and cultured overnight in Low calcium medium. The next day, keratinocytes were extensively washed in PBS 1× to remove dead cells and debris. Murine keratinocyte differentiation was induced by adding 1.2 mM CaCl_2_ to the growth medium of sub-confluent cells.

### 4.5. 12-O-tetradecanoylphorbol 13-acetate (TPA) Treatment

Application of 12-O-tetradecanoylphorbol 13-acetate (TPA) (Sigma-Aldrich, St. Louis, MO, USA, cat. num. P1585) dissolved in acetone (Sigma Aldrich, cat. num. 179124) was carried out on 6-week-old mouse back skin. The back hair was removed using an electric shaver 2 days before treatment. A total of 4 µg of TPA solution was applied once a day for 3 days on the dorsal skin of *hABCC1^flx/flx^* and knockout *hABCC1^−/−^* mice. Two hours after the final TPA application, animals were killed, and the skin samples were collected.

### 4.6. Protein Extraction and Immunoblotting Analysis

Immunoblot analyses were performed as previously described [61]. In detail, A253 cells were lysed with Triton buffer (50 mM Tris-HCl pH 7.5, 250 mM NaCl, 50 mM NaF, 1 mM EDTA pH 8, 0.1% Triton), plus protease inhibitors (cOmplete EDTA free protease inhibitor cocktail, Roche, Basel, Switzerland), PMSF, DTT and sodium orthovanadate (New England Biolabs, Ipswich, MA, USA). HEKn cells were lysed in SDS lysis buffer (100 mM Tris, pH8.8, 1%SDS, 5 mM EDTA, 20 mM DTT and 2 mM AEBSF). Proteins were separated by SDS/PAGE and transferred onto PVDF membranes. Membranes were blocked with 5% nonfat dry milk in PBS-T (phosphate-buffered saline and 0.1% Tween-20) for 1 h at room temperature (RT). Primary antibodies were incubated for 2 h at RT, followed by incubation with the appropriate horseradish peroxidase-conjugated secondary antibody ( anti-mouse cat. num. 170-5047, dilution: 1:10,000; anti-rabbit cat. num. 170-6515, dilution: 1:10,000; Bio-Rad, Hercules, CA, USA). Detection was performed with ECL chemiluminescence kit (Perkin-Elmer, Waltham, MA, USA).

The following antibodies were used: rabbit monoclonal anti p63-α (Cell Signaling Technology, Danvers, MA, USA, #13109 clone D2K8X, dilution: 1:1000), rabbit monoclonal anti-ABCC1 (Cell Signaling Technology, #72202 clone D5C1X, dilution: 1:1000), rabbit polyclonal anti-KRT10 (Covance PRB159P, dilution: 1:5000), mouse monoclonal anti β-actin (Sigma-Aldrich, #AC-15, dilution: 1:50,000), and mouse monoclonal anti-vinculin (Sigma-Aldrich #V9131, dilution: 1:50,000). Uncropped images related to the blots are shown in Figure S3.

### 4.7. Chromatin Immunoprecipitation (ChIP) Assay

HEKn cells were collected and fixed in 1% formaldehyde. Chromatin was sonicated into 200 to 500 bp fragments using a Diagenode Bioruptor. The chromatin immunoprecipitation was performed with an anti-p63 antibody (Cell Signaling Technology, #13109, clone D2K8X) or IgG (Invitrogen) as control, using the MAGnify Chromatin Immunoprecipitation System (Invitrogen, 492024). For the amplification of the intronic region containing potential p63 response elements, the following primers were used: Fw 5′-ATGGTGCTTGGGAGAGTTGG-3′; Rev 5′-CCACTGTGCCCAGTCCTAAG-3′. A negative control region was used to further confirm the specificity of the immunoprecipitation.

### 4.8. RNA Extraction and Real-Time PCR

Total RNA was purified utilizing the RNeasy mini kit (Qiagen, Hilden, Germany) following the manufacturer’s instructions, quantified by a NanoDrop Spectrophotometer (Thermo Scientific, Waltham, MA, USA) and retrotranscribed by SensiFast cDNA Synthesis (Bioline, London, UK). qPCR was performed using SYBR-Green PCR Master Mix (Thermo Scientific, cat. num. A25742), with the QuantStudio 5 Real-Time PCR Systems (Applied Biosystems, Waltham, MA, USA). The relative expression levels of each gene were calculated using the 2^−ΔΔCt^ method (Ct, threshold cycle). The following primers were used: hTBP fw 5′-CTGACAGGTAAGGAGGACGC-3′; hTBP rev 5′-AGTTACCTGACCTCTCCCCC-3′; hABCC1 fw 5′-TTACTCATTCAGCTCGTCTTGTC-3′; hABCC1 rev 5′-CAGGGATTAGGGTCGTGGAT-3′; h∆Np63 fw 5′-GAAGAAAGGACAGCAGCATTG-3′; h∆Np63 rev 5′-GGGACTGGTGGACGAGGAG-3′; mActin fw 5′-TGTCCCTGTATGCCTCTGGTC-3′; mActin rev 5′-GAACCGCTCGTTGCCAATAGT-3′.

### 4.9. Histological and Immunostaining Analysis

Cutaneous Squamous Cell Carcinoma TMA (SK802c) was purchased by US Biomax, Inc (Rockville, MD, USA). TMA slide includes 76 cases of squamous cell carcinoma, 2 each of adjacent normal skin tissue, plus 2 normal skin tissues.

Paraffin-embedded tissue (FFPE) sections (5 μm) were dewaxed, rehydrated and stained for 4 min with Mayer’s hematoxylin (Bio-Optica, Milano, Italy). After washing in distilled water, sections were incubated for 1 min in Eosin Y alcoholic solution (Bio-Optica), extensively washed in distilled water and finally dehydrated by 70, 90, 100% ethanol solution incubation. Immunohistochemistry (IHC) staining was used to analyze gene expression at the protein level. Deparaffinization of the FFPE sections (5 μm) was performed with Bioclear (Bio-Optica), with further rehydration in decreasing alcohol concentrations (absolute, 90, 70, 80 and 50% ethanol) and washing in distilled water. Antigen retrieval was performed by boiling in 0.01 M Sodium Citrate Buffer pH 6.0. The staining was performed in a serial manner as follows: primary antibody was incubated for 1 h in 5% goat serum (Gibco) in PBS at RT; immunoreactions continued using a DAB (3,3′-diaminobenzidine) Detection System (Histo-Line Laboratoires, Pantigliate, Italy, cat. num. CPH080) for secondary antibody detection, according to manufacturer’s protocols. Additional Mayer’s hematoxylin staining was performed. The following antibodies were used: mouse monoclonal anti p63-α (Abcam, Cambridge, UK; AB735, dilution: 1:100); rabbit monoclonal anti-ABCC1 (Cell Signaling Technology, clone D5C1X, dilution 1:75). The slides were then visualized using a Leica DM6 microscope (Leica Microsystems, Buccinasco, MI, Italy).

For the immunofluorescence staining, FFPE sections (5 μm) were dewaxed (Bio-Clear washing, Bio-Optica) and rehydrated by serial dilution of ethanol (100, 90, 80, 70 and 50% ethanol). Sections were boiled in 0.01 M Sodium Citrate Buffer pH 6.0 for antigen retrieval. For IF staining, we performed the following protocol. Sections were blocked for 1 h in 5% goat serum (Gibco) in PBS at RT. After blocking, sections were incubated with primary antibody for 1 h at RT and then with the appropriate secondary antibody for an additional hour at RT. Nuclei were counterstained with DAPI (4′,6-diamidino-2-phenylindole). The following antibodies were used: rabbit polyclonal anti-KRT10 (Covance PRB159P, dilution: 1:1000), mouse monoclonal anti-p63-α (Abcam AB735, clone 4A4, dilution: 1:100), mouse monoclonal anti-K14 (Abcam AB7800, clone LL002, dilution 1:1000), rabbit monoclonal anti-Ki67 (Cell Signaling Technology, Clone D3B65, dilution 1:200). Images were acquired by confocal microscope Leica Stellaris 8.

### 4.10. Bioinformatic Analysis

Gene expression data were downloaded from cBioportal [62,63,64] and UCSC Xena [65]. Correlation analyses were performed with R software version 4.3.1.

### 4.11. Statistical Analysis

The number of biological replicates is mentioned in the figure legends. The type of statistical test utilized to calculate the *p* value was reported in the figure legends.

## Data Availability

The original contributions presented in the study are included in the article/Supplementary Material, further inquiries can be directed to the corresponding authors.

## Supplementary Materials

The following supporting information can be downloaded at: https://www.mdpi.com/article/10.3390/ijms25168741/s1.

## References

[1] Melino G., Memmi E.M., Pelicci P.G., Bernassola F. (2015). Maintaining epithelial stemness with p63. Sci. Signal.

[2] Soares E., Zhou H. (2018). Master regulatory role of p63 in epidermal development and disease. Cell Mol. Life Sci..

[3] Osterburg C., Dotsch V. (2022). Structural diversity of p63 and p73 isoforms. Cell Death Differ..

[4] Trink B., Osada M., Ratovitski E., Sidransky D. (2007). p63 transcriptional regulation of epithelial integrity and cancer. Cell Cycle.

[5] Candi E., Cipollone R., Rivetti di Val Cervo P., Gonfloni S., Melino G., Knight R. (2008). p63 in epithelial development. Cell Mol. Life Sci..

[6] Li Y., Giovannini S., Wang T., Fang J., Li P., Shao C., Wang Y., Shi Y., Candi E., TOR centre (2023). p63: A crucial player in epithelial stemness regulation. Oncogene.

[7] Yang A., Schweitzer R., Sun D., Kaghad M., Walker N., Bronson R.T., Tabin C., Sharpe A., Caput D., Crum C. (1999). p63 is essential for regenerative proliferation in limb, craniofacial and epithelial development. Nature.

[8] Mills A.A., Zheng B., Wang X.J., Vogel H., Roop D.R., Bradley A. (1999). p63 is a p53 homologue required for limb and epidermal morphogenesis. Nature.

[9] Romano R.A., Smalley K., Magraw C., Serna V.A., Kurita T., Raghavan S., Sinha S. (2012). DeltaNp63 knockout mice reveal its indispensable role as a master regulator of epithelial development and differentiation. Development.

[10] Kouwenhoven E.N., Oti M., Niehues H., van Heeringen S.J., Schalkwijk J., Stunnenberg H.G., van Bokhoven H., Zhou H. (2015). Transcription factor p63 bookmarks and regulates dynamic enhancers during epidermal differentiation. EMBO Rep..

[11] Kouwenhoven E.N., van Bokhoven H., Zhou H. (2015). Gene regulatory mechanisms orchestrated by p63 in epithelial development and related disorders. Biochim. Biophys. Acta.

[12] Carroll D.K., Carroll J.S., Leong C.O., Cheng F., Brown M., Mills A.A., Brugge J.S., Ellisen L.W. (2006). p63 regulates an adhesion programme and cell survival in epithelial cells. Nat. Cell Biol..

[13] Fisher M.L., Balinth S., Mills A.A. (2020). p63-related signaling at a glance. J. Cell Sci..

[14] Westfall M.D., Mays D.J., Sniezek J.C., Pietenpol J.A. (2003). The Delta Np63 alpha phosphoprotein binds the p21 and 14-3-3 sigma promoters in vivo and has transcriptional repressor activity that is reduced by Hay-Wells syndrome-derived mutations. Mol. Cell Biol..

[15] Sen G.L., Boxer L.D., Webster D.E., Bussat R.T., Qu K., Zarnegar B.J., Johnston D., Siprashvili Z., Khavari P.A. (2012). ZNF750 is a p63 target gene that induces KLF4 to drive terminal epidermal differentiation. Dev. Cell.

[16] Candi E., Rufini A., Terrinoni A., Giamboi-Miraglia A., Lena A.M., Mantovani R., Knight R., Melino G. (2007). DeltaNp63 regulates thymic development through enhanced expression of FgfR2 and Jag2. Proc. Natl. Acad. Sci. USA.

[17] Fessing M.Y., Mardaryev A.N., Gdula M.R., Sharov A.A., Sharova T.Y., Rapisarda V., Gordon K.B., Smorodchenko A.D., Poterlowicz K., Ferone G. (2011). p63 regulates Satb1 to control tissue-specific chromatin remodeling during development of the epidermis. J. Cell Biol..

[18] LeBoeuf M., Terrell A., Trivedi S., Sinha S., Epstein J.A., Olson E.N., Morrisey E.E., Millar S.E. (2010). Hdac1 and Hdac2 act redundantly to control p63 and p53 functions in epidermal progenitor cells. Dev. Cell.

[19] Mardaryev A.N., Gdula M.R., Yarker J.L., Emelianov V.U., Poterlowicz K., Sharov A.A., Sharova T.Y., Scarpa J.A., Joffe B., Solovei I. (2014). p63 and Brg1 control developmentally regulated higher-order chromatin remodelling at the epidermal differentiation complex locus in epidermal progenitor cells. Development.

[20] Yi M., Tan Y., Wang L., Cai J., Li X., Zeng Z., Xiong W., Li G., Li X., Tan P. (2020). TP63 links chromatin remodeling and enhancer reprogramming to epidermal differentiation and squamous cell carcinoma development. Cell Mol. Life Sci..

[21] Fierro C., Gatti V., La Banca V., De Domenico S., Scalera S., Corleone G., Fanciulli M., De Nicola F., Mauriello A., Montanaro M. (2023). The long non-coding RNA NEAT1 is a DeltaNp63 target gene modulating epidermal differentiation. Nat. Commun..

[22] Gatti V., Fierro C., Annicchiarico-Petruzzelli M., Melino G., Peschiaroli A. (2019). DeltaNp63 in squamous cell carcinoma: Defining the oncogenic routes affecting epigenetic landscape and tumour microenvironment. Mol. Oncol..

[23] Gatti V., Bongiorno-Borbone L., Fierro C., Annicchiarico-Petruzzelli M., Melino G., Peschiaroli A. (2019). p63 at the Crossroads between Stemness and Metastasis in Breast Cancer. Int. J. Mol. Sci..

[24] Fisher M.L., Balinth S., Mills A.A. (2023). DeltaNp63alpha in cancer: Importance and therapeutic opportunities. Trends Cell Biol..

[25] Rothenberg S.M., Ellisen L.W. (2012). The molecular pathogenesis of head and neck squamous cell carcinoma. J. Clin. Investig..

[26] Agrawal N., Frederick M.J., Pickering C.R., Bettegowda C., Chang K., Li R.J., Fakhry C., Xie T.X., Zhang J., Wang J. (2011). Exome sequencing of head and neck squamous cell carcinoma reveals inactivating mutations in NOTCH1. Science.

[27] Saladi S.V., Ross K., Karaayvaz M., Tata P.R., Mou H., Rajagopal J., Ramaswamy S., Ellisen L.W. (2017). ACTL6A Is Co-Amplified with p63 in Squamous Cell Carcinoma to Drive YAP Activation, Regenerative Proliferation, and Poor Prognosis. Cancer Cell.

[28] Maier S., Wilbertz T., Braun M., Scheble V., Reischl M., Mikut R., Menon R., Nikolov P., Petersen K., Beschorner C. (2011). SOX2 amplification is a common event in squamous cell carcinomas of different organ sites. Hum. Pathol..

[29] Botti E., Spallone G., Moretti F., Marinari B., Pinetti V., Galanti S., De Meo P.D., De Nicola F., Ganci F., Castrignano T. (2011). Developmental factor IRF6 exhibits tumor suppressor activity in squamous cell carcinomas. Proc. Natl. Acad. Sci. USA.

[30] Yang X., Lu H., Yan B., Romano R.A., Bian Y., Friedman J., Duggal P., Allen C., Chuang R., Ehsanian R. (2011). DeltaNp63 versatilely regulates a Broad NF-kappaB gene program and promotes squamous epithelial proliferation, migration, and inflammation. Cancer Res..

[31] Compagnone M., Gatti V., Presutti D., Ruberti G., Fierro C., Markert E.K., Vousden K.H., Zhou H., Mauriello A., Anemone L. (2017). DeltaNp63-mediated regulation of hyaluronic acid metabolism and signaling supports HNSCC tumorigenesis. Proc. Natl. Acad. Sci. USA.

[32] Gatti V., Fierro C., Compagnone M., Giangrazi F., Markert E.K., Bongiorno-Borbone L., Melino G., Peschiaroli A. (2018). DeltaNp63 regulates the expression of hyaluronic acid-related genes in breast cancer cells. Oncogenesis.

[33] Moses M.A., George A.L., Sakakibara N., Mahmood K., Ponnamperuma R.M., King K.E., Weinberg W.C. (2019). Molecular Mechanisms of p63-Mediated Squamous Cancer Pathogenesis. Int. J. Mol. Sci..

[34] Leslie E.M., Deeley R.G., Cole S.P. (2001). Toxicological relevance of the multidrug resistance protein 1, MRP1 (ABCC1) and related transporters. Toxicology.

[35] Mohle L., Stefan K., Bascunana P., Brackhan M., Bruning T., Eiriz I., El Menuawy A., van Genderen S., Santos-Garcia I., Gorska A.M. (2023). ABC Transporter C1 Prevents Dimethyl Fumarate from Targeting Alzheimer’s Disease. Biology.

[36] Krohn M., Lange C., Hofrichter J., Scheffler K., Stenzel J., Steffen J., Schumacher T., Bruning T., Plath A.S., Alfen F. (2011). Cerebral amyloid-beta proteostasis is regulated by the membrane transport protein ABCC1 in mice. J. Clin. Investig..

[37] Gatti V., Fierro C., Compagnone M., La Banca V., Mauriello A., Montanaro M., Scalera S., De Nicola F., Candi E., Ricci F. (2022). DeltaNp63-Senataxin circuit controls keratinocyte differentiation by promoting the transcriptional termination of epidermal genes. Proc. Natl. Acad. Sci. USA.

[38] Gatti V., Bernassola F., Talora C., Melino G., Peschiaroli A. (2020). The Impact of the Ubiquitin System in the Pathogenesis of Squamous Cell Carcinomas. Cancers.

[39] Zeng W., Xie F., Pan Y., Chen Z., Chen H., Liu X., Tian K., Xu D. (2023). A comprehensive prognostic score for head and neck squamous cancer driver genes and phenotype traits. Discov. Oncol..

[40] Corchado-Cobos R., Garcia-Sancha N., Gonzalez-Sarmiento R., Perez-Losada J., Canueto J. (2020). Cutaneous Squamous Cell Carcinoma: From Biology to Therapy. Int. J. Mol. Sci..

[41] Winge M.C.G., Kellman L.N., Guo K., Tang J.Y., Swetter S.M., Aasi S.Z., Sarin K.Y., Chang A.L.S., Khavari P.A. (2023). Advances in cutaneous squamous cell carcinoma. Nat. Rev. Cancer.

[42] Deeley R.G., Westlake C., Cole S.P. (2006). Transmembrane transport of endo- and xenobiotics by mammalian ATP-binding cassette multidrug resistance proteins. Physiol. Rev..

[43] Hanssen K.M., Haber M., Fletcher J.I. (2021). Targeting multidrug resistance-associated protein 1 (MRP1)-expressing cancers: Beyond pharmacological inhibition. Drug Resist. Updat..

[44] Robey R.W., Pluchino K.M., Hall M.D., Fojo A.T., Bates S.E., Gottesman M.M. (2018). Revisiting the role of ABC transporters in multidrug-resistant cancer. Nat. Rev. Cancer.

[45] Cole S.P., Sparks K.E., Fraser K., Loe D.W., Grant C.E., Wilson G.M., Deeley R.G. (1994). Pharmacological characterization of multidrug resistant MRP-transfected human tumor cells. Cancer Res..

[46] Allen J.D., Brinkhuis R.F., van Deemter L., Wijnholds J., Schinkel A.H. (2000). Extensive contribution of the multidrug transporters P-glycoprotein and Mrp1 to basal drug resistance. Cancer Res..

[47] Nedeljkovic M., Tanic N., Prvanovic M., Milovanovic Z., Tanic N. (2021). Friend or foe: ABCG2, ABCC1 and ABCB1 expression in triple-negative breast cancer. Breast Cancer.

[48] Takebayashi Y., Akiyama S., Natsugoe S., Hokita S., Niwa K., Kitazono M., Sumizawa T., Tani A., Furukawa T., Aikou T. (1998). The expression of multidrug resistance protein in human gastrointestinal tract carcinomas. Cancer.

[49] Tsuzuki H., Fujieda S., Sunaga H., Sugimoto C., Tanaka N., Saito H. (1998). Expression of multidrug resistance-associated protein (MRP) in head and neck squamous cell carcinoma. Cancer Lett..

[50] Larbcharoensub N., Leopairat J., Sirachainan E., Narkwong L., Bhongmakapat T., Rasmeepaisarn K., Janvilisri T. (2008). Association between multidrug resistance-associated protein 1 and poor prognosis in patients with nasopharyngeal carcinoma treated with radiotherapy and concurrent chemotherapy. Hum. Pathol..

[51] Eijdems E.W., De Haas M., Coco-Martin J.M., Ottenheim C.P., Zaman G.J., Dauwerse H.G., Breuning M.H., Twentyman P.R., Borst P., Baas F. (1995). Mechanisms of MRP over-expression in four human lung-cancer cell lines and analysis of the MRP amplicon. Int. J. Cancer.

[52] Ji L., Li H., Gao P., Shang G., Zhang D.D., Zhang N., Jiang T. (2013). Nrf2 pathway regulates multidrug-resistance-associated protein 1 in small cell lung cancer. PLoS ONE.

[53] Kurz E.U., Cole S.P., Deeley R.G. (2001). Identification of DNA-protein interactions in the 5′ flanking and 5′ untranslated regions of the human multidrug resistance protein (MRP1) gene: Evaluation of a putative antioxidant response element/AP-1 binding site. Biochem. Biophys. Res. Commun..

[54] Manohar C.F., Bray J.A., Salwen H.R., Madafiglio J., Cheng A., Flemming C., Marshall G.M., Norris M.D., Haber M., Cohn S.L. (2004). MYCN-mediated regulation of the MRP1 promoter in human neuroblastoma. Oncogene.

[55] Si X., Gao Z., Xu F., Zheng Y. (2020). SOX2 upregulates side population cells and enhances their chemoresistant ability by transactivating ABCC1 expression contributing to intrinsic resistance to paclitaxel in melanoma. Mol. Carcinog..

[56] Jiang Y., Jiang Y.Y., Xie J.J., Mayakonda A., Hazawa M., Chen L., Xiao J.F., Li C.Q., Huang M.L., Ding L.W. (2018). Co-activation of super-enhancer-driven CCAT1 by TP63 and SOX2 promotes squamous cancer progression. Nat. Commun..

[57] Minich T., Riemer J., Schulz J.B., Wielinga P., Wijnholds J., Dringen R. (2006). The multidrug resistance protein 1 (Mrp1), but not Mrp5, mediates export of glutathione and glutathione disulfide from brain astrocytes. J. Neurochem..

[58] Koontongkaew S. (2013). The tumor microenvironment contribution to development, growth, invasion and metastasis of head and neck squamous cell carcinomas. J. Cancer.

[59] Liu S., Wang R., Fang J. (2024). Exploring the frontiers: Tumor immune microenvironment and immunotherapy in head and neck squamous cell carcinoma. Discov. Oncol..

[60] Krohn M., Zoufal V., Mairinger S., Wanek T., Paarmann K., Brüning T., Eiriz I., Brackhan M., Langer O., Pahnke J. (2019). Generation and Characterization of an Abcc1 Humanized Mouse Model (hABCC1(flx/flx)) with Knockout Capability. Mol. Pharmacol..

[61] Malatesta M., Peschiaroli A., Memmi E.M., Zhang J., Antonov A., Green D.R., Barlev N.A., Garabadgiu A.V., Zhou P., Melino G. (2013). The Cul4A-DDB1 E3 ubiquitin ligase complex represses p73 transcriptional activity. Oncogene.

[62] Cerami E., Gao J., Dogrusoz U., Gross B.E., Sumer S.O., Aksoy B.A., Jacobsen A., Byrne C.J., Heuer M.L., Larsson E. (2012). The cBio cancer genomics portal: An open platform for exploring multidimensional cancer genomics data. Cancer Discov..

[63] Gao J., Aksoy B.A., Dogrusoz U., Dresdner G., Gross B., Sumer S.O., Sun Y., Jacobsen A., Sinha R., Larsson E. (2013). Integrative analysis of complex cancer genomics and clinical profiles using the cBioPortal. Sci. Signal.

[64] de Bruijn I., Kundra R., Mastrogiacomo B., Tran T.N., Sikina L., Mazor T., Li X., Ochoa A., Zhao G., Lai B. (2023). Analysis and Visualization of Longitudinal Genomic and Clinical Data from the AACR Project GENIE Biopharma Collaborative in cBioPortal. Cancer Res..

[65] Goldman M.J., Craft B., Hastie M., Repecka K., McDade F., Kamath A., Banerjee A., Luo Y., Rogers D., Brooks A.N. (2020). Visualizing and interpreting cancer genomics data via the Xena platform. Nat. Biotechnol..

