# Supplementary material for: ABCC1 Is a ΔNp63 Target Gene Overexpressed in Squamous Cell Carcinoma
Source: Int J Mol Sci. 2024 Aug 10;25(16):8741. doi: 10.3390/ijms25168741 (PMC11354449; doi:10.3390/ijms25168741)
Supplement: Supplementary file 1 [file ijms-25-08741-s001.zip › ijms-3113843-supplementary.pdf]

Supplementary Information for "*ABCC1 is a ΔNp63 target gene overexpressed in squamous cell carcinoma*"

Corresponding author:

Angelo Peschiaroli, angelo.peschiaroli@cnr.it

Sivia D'Amico, silvia.damico@ift.cnr.it

This Supplementary information includes:

Figures S1-S3 with legends

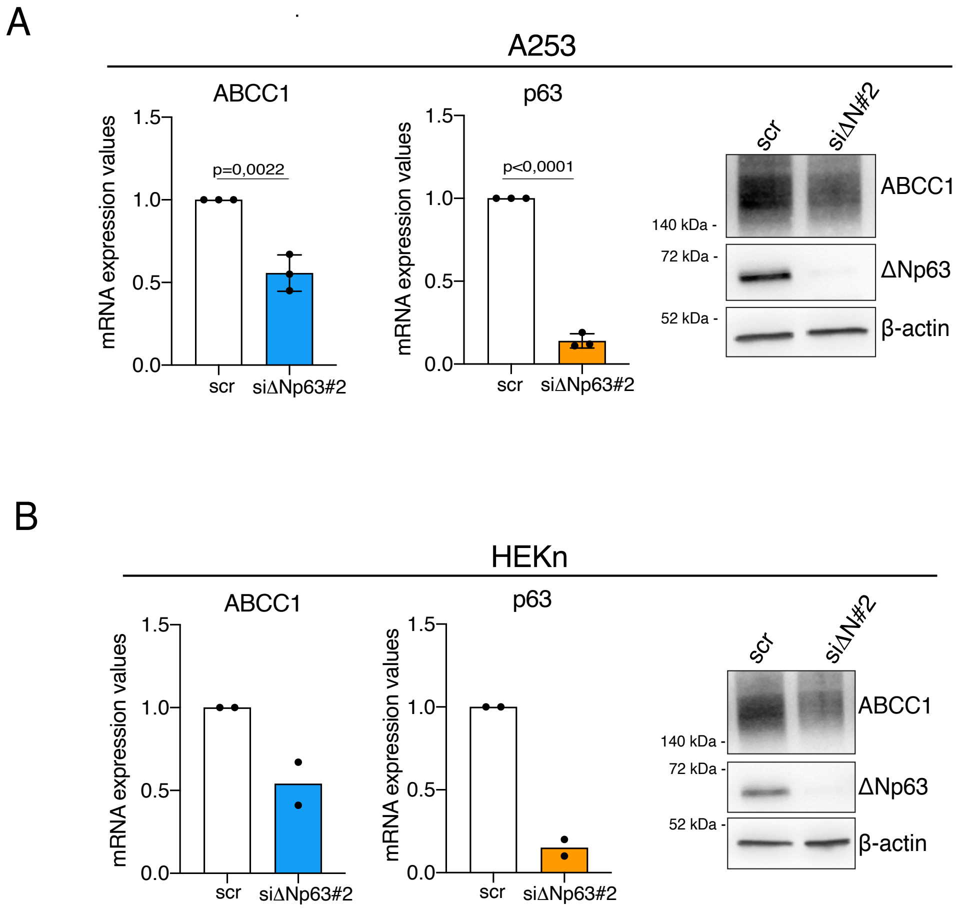

Figure S1. A) RT-qPCR analysis (left panels) of ABCC1 and p63 mRNA levels in A253 transfected with siRNA targeting ΔNp63 isoform (siΔNp63#2) or non-relevant mRNA (scr). The mean of three (n=3) independent biological replicates ± SD is shown. p value was calculated using two-tailed unpaired Student's t test. In parallel, western blotting analysis using antibodies to the indicated proteins (right panel) was performed utilizing protein lysates from transfected cells. B) RT-qPCR analysis of ABCC1 and p63 mRNA levels (left panels) in human primary keratinocytes (HEKn) transfected as in A). The mean of two (n=2) independent biological replicates is shown. In parallel, western blotting analysis using antibodies to the indicated proteins (right panel) was performed utilizing protein lysates from transfected cells.

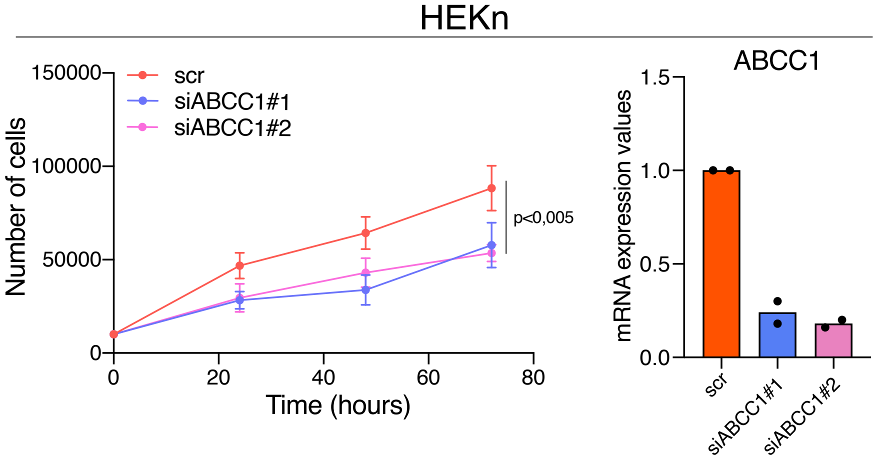

Figure S2. Human primary keratinocytes (HEKn) were transfected with control oligos (scr) and two different ABCC1 targeting siRNA oligos (siABCC1#1 and siABCC1#2). 24 hours post transfection transfected cells were replated and cell number and viability were assessed by trypan blu exclusion over a period of 72 hours (left panel). p value was calculated using two-tailed unpaired Student's t test. The efficiency of ABCC1 silencing was determined by RT-qPCR analysis performed 48 hours post transfection (left panel). The mean of two (n=2) independent biological replicates is shown

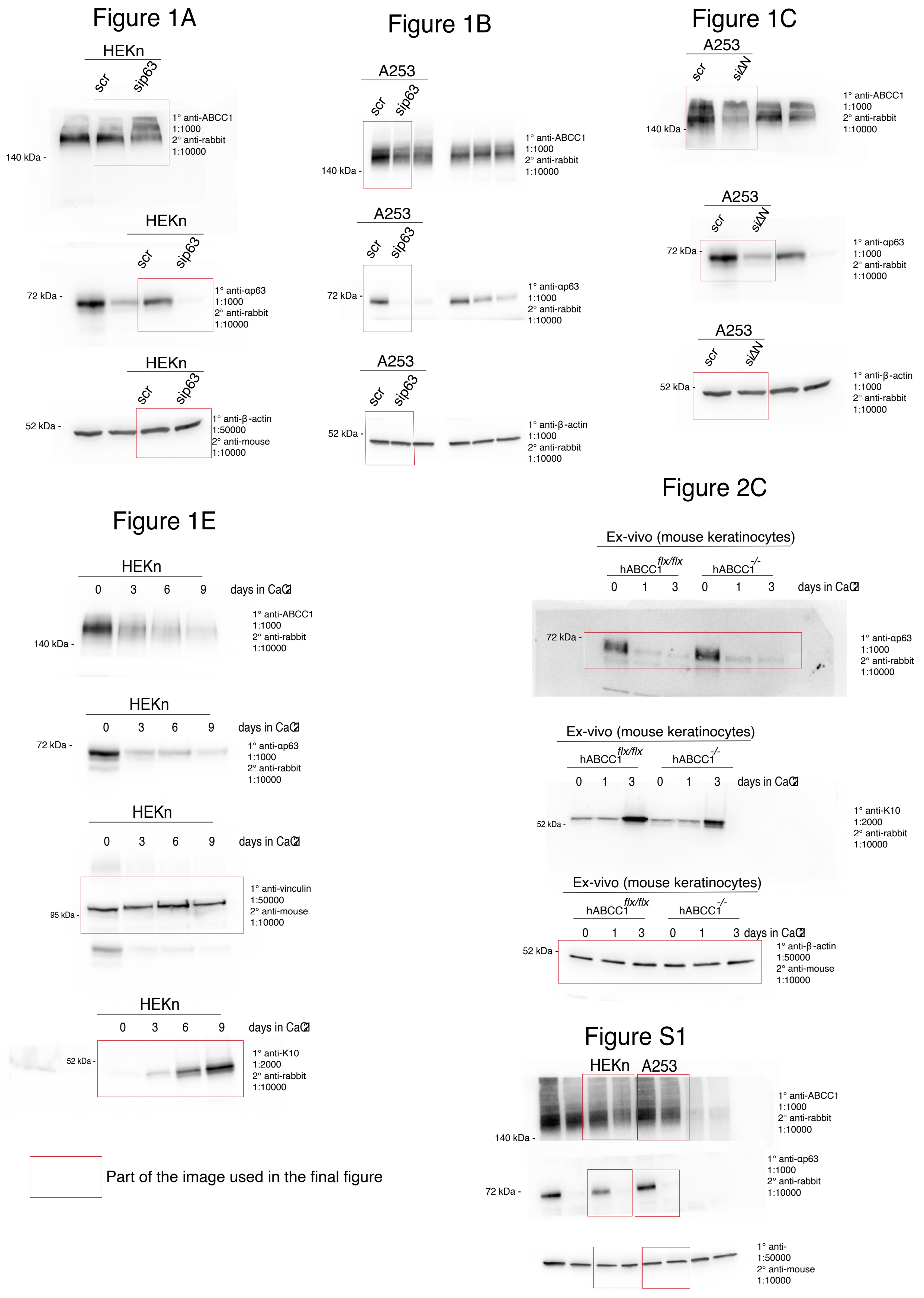

Figure S3. Uncropped immunoblotting images.
